# A commonly used anti-SOX15 antibody fails to demonstrate specificity in mouse embryos.

**DOI:** 10.17912/micropub.biology.001558

**Published:** 2025-03-18

**Authors:** Farina Aziz, Bin Gu, Amy Ralston

**Affiliations:** 1 Cell and Molecular Biology Program, Michigan State University, East Lansing, MI, USA; 2 Department of Obstetrics, Gynecology and Reproductive Biology, Michigan State University, East Lansing, MI, USA; 3 Department of Biochemistry and Molecular Biology, Michigan State University, East Lansing, MI, USA

## Abstract

SOX15 is a broadly conserved transcription factor involved in many critical processes, including mammalian cell fate specification and pluripotency. We investigated the specificity of a commercially available, polyclonal anti-SOX15 antibody advertised as knockout-validated. We generated a new mouse line carrying a null allele of *Sox15* , and then evaluated anti-SOX15 activity in *Sox15* null mouse embryos. Nuclear signal was detected in both wild type and null embryos, even when potential maternally or paternally-contributed *Sox15* was eliminated. We conclude that this SOX15-detecting reagent may not be suitable for all applications, and caution the growing community of users accordingly.

**Figure 1. A commonly used anti-SOX15 antibody fails to demonstrate specificity in mouse embryos.**
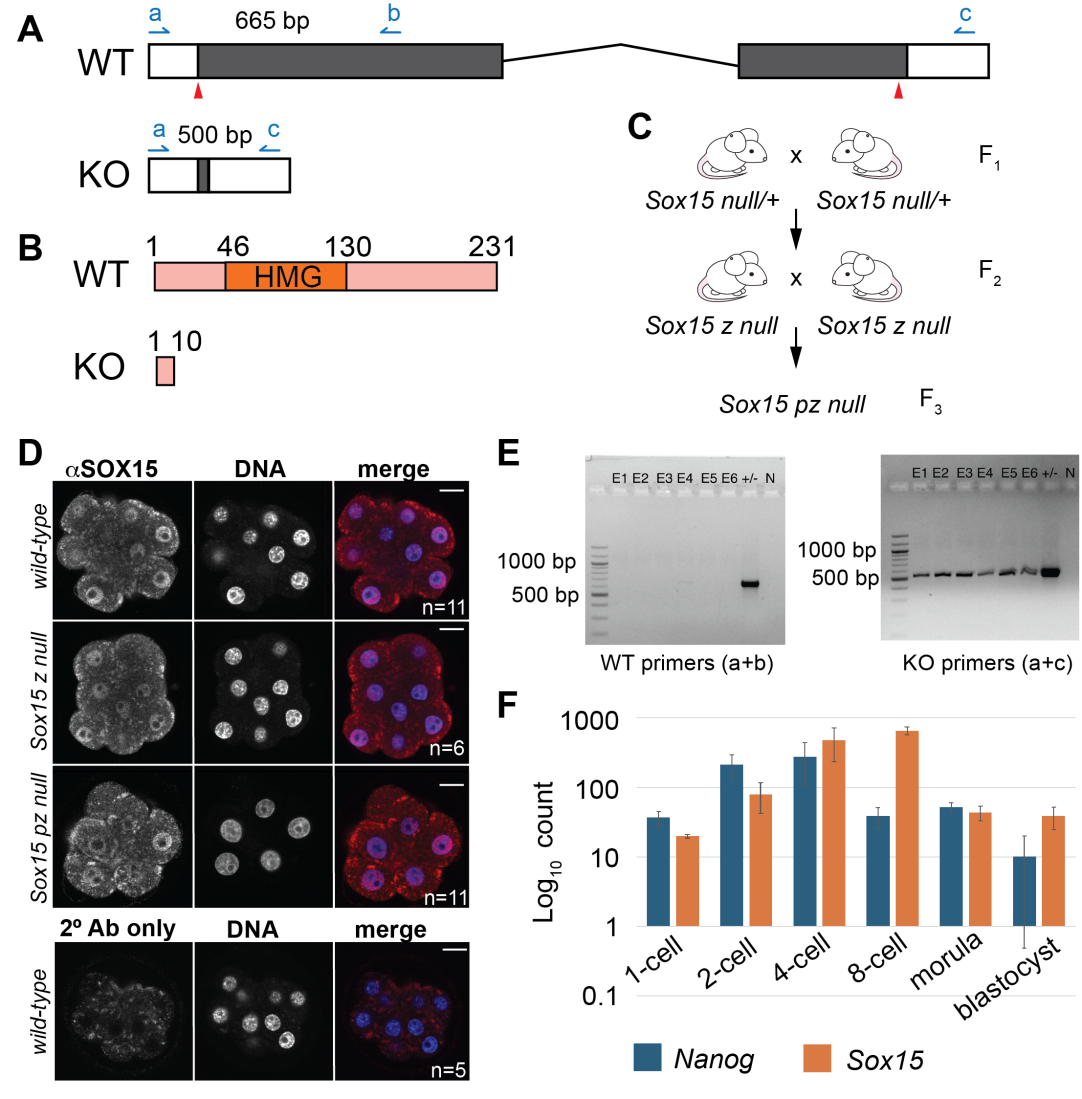
A) The wild type (WT) *Sox15* locus was targeted for CRISPR/CAS9 deletion using sgRNAs targeting exons 1 and 2 *Sox15* (red arrowheads). Locations of genotyping primers (a,b,c) and predicted PCR product sizes are shown.Successful deletion would result in a ~1100 base pair (bp) deletion knockout (KO) allele. B) Schematic representation of 231 amino acid (aa) WT SOX15 protein, with DNA-binding HMG domain highlighted, and 10 aa predicted SOX15 KO fragment. C) Crosses used to generate embryos examined in Panels D, E. D) Representative confocal sections of mouse morula stage (E3.25) embryos immunofluorescently stained using anti-SOX15 (25415-1-AP) of indicated genotypes (z = zygotic, p = parental). A persistent nuclear signal is detected in the absence of *Sox15 .* No signal is detected when only the secondary antibody was applied. Bar = 20 µm E) PCR confirmation of genotypes for embryos (E1-E6), including those shown in Panel D. Heterozygous ear punch DNA (+/-) is shown as positive control. Sample N lacked added DNA). F) Expression levels of *Nanog* and *Sox15* mRNA throughout preimplantation; published data (Xie et al., 2010).

## Description

SOX proteins are SRY-related HMG-box transcription factors that play essential roles in many biological contexts (Kamachi & Kondoh, 2013; Sarkar & Hochedlinger, 2013). The Group G SOX encoded by *Sox15* has been implicated in placental development (Ito, 2010; Yamada et al., 2008), neurogenesis (Choi et al., 2023), muscle regeneration (Lee et al., 2004; Savage et al., 2009) and cancer (Hu et al., 2024; Moradi et al., 2017). Additionally, SOX15 expression is enriched in mouse embryonic stem (ES) cells, where it helps promote pluripotency (Choi et al., 2023; Maruyama et al., 2005). ES cells are derived from the mouse embryo during preimplantation, when pluripotency is first established. Yet the SOX15 expression pattern in preimplantation mouse embryos has not been reported.

To investigate SOX15 expression in mouse embryos, we selected an anti-SOX15 antibody that had previously been knockout-validated in mouse ES cells (Choi et al., 2023). That is, a protein of the predicted size of SOX15 was detected in wild type mouse ES cells by Western blot using a polyclonal antibody (25415-1-AP) raised against amino acids 122-231 of SOX15, but not in ES cells carrying two alleles encoding the N-terminal 72 amino acids of SOX15. To ensure that we would also be able to validate this antibody in embryos, we generated a new mouse line carrying a *Sox15* knockout allele, predicted to encode the first 10 amino acids of *Sox15* ( Fig. 1A, B), by CRISPR/Cas9-mediated genome editing.

We then bred mice carrying the *Sox15* knockout allele to generate preimplantation embryos that were lacking zygotically transcribed *Sox15* ( Fig. 1C ). Because homozygous *Sox15* knockout mice are viable and fertile (Lee et al., 2004; Maruyama et al., 2005), we also generated *Sox15* null adults. These *Sox15* null adults were subsequently intercrossed to produce embryos lacking *Sox15* mRNA or protein potentially carried by gametes, as well as embryo-expressed *Sox15* .

We then evaluated anti-SOX15 activity in preimplantation embryos by immunofluorescence and confocal microscopy ( Fig. 1D, E). During preimplantation, *Sox15* mRNA is at least as abundant as *Nanog* ( Fig. 1F ), and NANOG is robustly detected by immunofluorescence during preimplantation (Strumpf et al., 2005). This provided confidence that SOX15 should be detectable by immunofluorescence during preimplantation. In fact, the anti-SOX15 antibody detected a nuclear signal in wild type embryos ( Fig. 1D ). However, this signal persisted with no apparent reduction in *Sox15* null embryos, indicating that the antibody fails to accurately and specifically detect SOX15 in mouse embryos.

This finding underscores the need for characterization of antibody performance in specific applications (Kahn et al., 2024). We note that lot-to-lot variation could also impact results, but lot numbers have not been reported in publications that may have used this reagent for immunofluorescence or other application (Belair-Hickey et al., 2024; Choi et al., 2023; Hu et al., 2024; Liu et al., 2022). Increased transparency and reporting standards during publishing could positively impact the rigor of antibody-dependent studies in general (Taussig et al., 2018). Databases, technologies, and high throughput strategies to curtail the “antibody characterization crisis” have also been described (Ayoubi et al., 2024; Kahn et al., 2024). Ultimately, to ensure accurate characterization of SOX15, future studies should incorporate alternative well-characterized antibodies or orthogonal detection methods, such as knock-in reporters or proteomic approaches.

## Methods

All animal research was conducted in accordance with the guidelines of the Michigan State University Institutional Animal Care and Use Committee. Mice were maintained on a 12 hr light/dark cycle.

The *Sox15* null allele was generated by injecting sgRNAs and CAS9 enzyme sgRNAs into the pronucleus of zygotes and then transferring to recipient females following standard practices.

Mouse genotypes were determined by PCR using genomic DNA extracted from ear biopsy using the REDExtract-N-Amp kit. Embryo genomic DNA was extracted using the same kit, in 10 µl total volume. Genomic extracts (1-2 µl) were then subjected to PCR using allele-specific primers. PCR conditions were: 95 °C for 5 minutes, 30 cycles of 95 °C for 30 seconds, 57 °C for 1 minute, 68 °C (30 seconds KO and 40 seconds WT), and 68 °C for 5 minutes.

Embryos were collected by flushing the oviduct or uterus with M2 medium. Embryos were then incubated in 4% formaldehyde for 10 minutes at room temperature, 0.5% Triton X-100 in PBS for 30 minutes at room temperature, blocking solution (10% Fetal Bovine Serum, 0.1% Triton X-100 in PBS) overnight at 4 °C, primary antibody (anti-rabbit anti-SOX15, 1:400 in blocking solution) overnight at 4 ºC, blocking solution for 30 minutes at room temperature, 2º antibody (Donkey-anti-rabbit IgG Alexa 546, 1:400 in blocking solution) for 1 hour, blocking solution for 30 minutes at room temperature, and finally imaged in DRAQ5 1:400 in blocking solution.

Embryos were imaged using an Olympus FluoView FV1000 Confocal Laser Scanning Microscope system with 60× PlanApoN oil (NA 1.42) objective. For each embryo, *z* -stacks were collected, with 5 µm intervals between optical sections. All embryos were imaged before knowledge of their genotypes. Genotypes were determined by PCR after imaging as described above.

## Reagents

CD-1 mice (Jackson Labs)

Fetal Bovine Serum (Hyclone)

M2 Medium (Millipore)

Formaldehyde (Polysciences)

Triton X-100 (Sigma Aldrich)

PBS (Sigma Aldrich)

DRAQ5 (Cell Signaling Technologies)

Rabbit anti-SOX15 (25415-1-AP, ProteinTech, lot 00082448)

Donkey anti-rabbit IgG Alexa 546 (Invitrogen, A10040)

REDExtract-N-Amp Kit (Sigma-Aldrich)

sgRNAs (Synthego): GCTCCTCACAAGCAGAGACT, AAGGGAAGTATTATATGGAG

Allele-specific primers (IDT): a) GCAGCTGTTGGGACTTTGTG, b) TCGGTACTTGTAGTCGGGATAG, c) GTTGCTGCTGTAGGGAGAGAATAC (see Fig. 1).
